# Per-oral endoscopic tunneling transgastric drainage of an acute pancreatic necrotic collection

**DOI:** 10.1055/a-2759-2586

**Published:** 2026-02-20

**Authors:** Anastasios C. Manolakis, Tryfonas Mpektsis, Konstantinos Argyriou, Eirini Deligianni, Dimitrios Chougias, Ashish Sharma, Andreas Kapsoritakis

**Affiliations:** 1 393317 University of Thessaly School of Medicine, Larisa, Greece; 2 Department of Gastroenterology, University Hospital of Larissa, Larissa, Greece; 3 6932 Center for Advanced Therapeutic Endoscopy, University of Rochester, Rochester, New York, United States

Acute pancreatic necrotic collections (APNCs) complicating necrotic pancreatitis are managed conservatively. Upon onset of complications, percutaneous, endoscopic ultrasound (EUS)-guided or surgical drainage can be applied 1 2 .

A 40-year-old with severe necrotic pancreatitis became critically ill on week 3 due to an infected APNC, diagnosed via computed tomography (CT). Percutaneous and EUS-drainage failed while surgery carried a high risk of fatal outcomes. During gastroscopy, no visible “bulge” corresponding to a compression or indentation from the collection was identified. Based on anatomy and CT, the left lateral–posterior wall of the corpus–antrum junction below the incisura exhibited optimal APNC-gastric wall contact ( Fig. 1 ). A novel endoscopic technique conforming to the principles of NOTES, termed per-oral endoscopic tunneling transgastric drainage (POET-D), was applied as a rescue therapy ( Video 1 ).

**Fig. 1:**
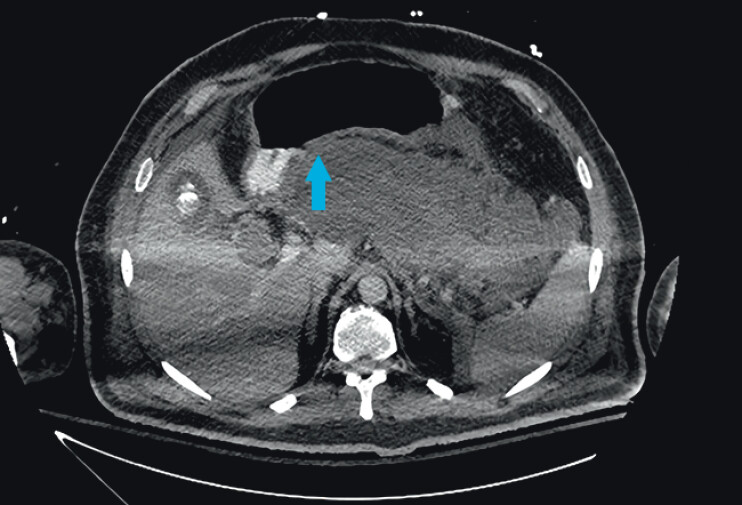
Computed tomography image showing the site of optimal contact (blue arrow) between the pancreatic collection and the gastric wall.

Per-oral endoscopic tunneling transgastric drainage (POET-D) of an acute pancreatic necrotic collection.Video 1

First, submucosal injection of 10 cc indigo carmine-solution was performed at the left lateral–posterior wall of the corpus–antrum junction. A mucosal incision was made using an endoscopic knife. A short submucosal tunnel was created. Muscle fibers and serosa were gradually dissected near the distal end of the tunnel to allow for the preservation of a mucosal flap above the distal defect. The endoscope entered the omental bursa. The omentum, spleen and splenic ligament could be visualized. 560 cc of Klebsiella spp-infected APNCs were aspirated and the cavity was lavaged with saline and gentamycin. A nasobiliary tube was placed inside the omental bursa for further drainage and lavage. Within 48 hours the patient improved. The tube was removed and the defect was closed with clips.

POET-D allows for preservation of a mucosal flap over the sero-muscular defect and continuous visualization of adjacent structures during dissection. It can provide rapid, large-volume drainage of viscous contents and lavage of infected cavities. POET-D can treat complicated cases of APNCs, potentially filling a therapeutic gap in-between percutaneous, EUS and surgical procedures.

Endoscopy_UCTN_Code_TTT_1AO_2AG_3AZ

Citation Format

Endoscopy 2025; 57: E1165–E1166. DOI: 10.1055/a-2715-4294

## Notes

Endoscopy E-Videos https://eref.thieme.de/e-videos
*E-Videos* is an open access online section of the journal *Endoscopy* , reporting on interesting cases and new techniques in gastroenterological endoscopy. All papers include a high-quality video and are published with a Creative Commons CC-BY license. Endoscopy E-Videos qualify for HINARI discounts and waivers and eligibility is automatically checked during the submission process. We grant 100% waivers to articles whose corresponding authors are based in Group A countries and 50% waivers to those who are based in Group B countries as classified by Research4Life (see: https://www.research4life.org/access/eligibility/ ). This section has its own submission website at https://mc.manuscriptcentral.com/e-videos .

## References

[1] Baron TH DiMaio CJ Wang AY American Gastroenterological Association Clinical Practice Update: Management of Pancreatic Necrosis Gastroenterology 2020 158 67 75 10.1053/j.gastro.2019.07.064 31479658

[2] Arvanitakis M Dumonceau JM Albert J Endoscopic management of acute necrotizing pancreatitis: European Society of Gastrointestinal Endoscopy (ESGE) evidence-based multidisciplinary guidelines Endoscopy 2018 50 524 546 10.1055/a-0588-5365 29631305

